# The Dawning Era of Personalized Medicine Exposes a Gap in Medical Education

**DOI:** 10.1371/journal.pmed.1000138

**Published:** 2009-08-25

**Authors:** Keyan Salari

**Affiliations:** Department of Genetics, Stanford University, Stanford, California, United States of America

## Abstract

Medical student Keyan Salari argues that it is crucial that medical students be trained to use and interpret patients' genetic information appropriately and responsibly.

As personal genetic information becomes an increasingly frequent component of the patient medical record, it is crucial that medical students be trained to use and interpret this information appropriately and responsibly. Here, I argue the need for medical education reform that equips physicians with the knowledge, skills, and attitudes required to practice personalized medicine.

## The Era of Personalized Medicine

The sequencing of the human genome, followed by the related HapMap project, and the explosive number of genome-wide association studies conducted over the last decade, have heralded a new era of medicine. The vision of a *personalized medicine*, where a patient's personal genetic and environmental information is used collectively to predict individual risks of disease and responsiveness to drugs, promises to revolutionize the medical management of many illnesses. Physicians have long used environmental factors like diet and exercise in preventative health and treatment strategies; however, despite being championed by genome scientists and those in the biotechnology industry, personalized medicine has yet to be adopted by most clinicians. It has, however, already reached a growing number of consumers, who are helping to usher in this new era as they arrive at their doctor's office with their personal genetic code and a long list of questions in hand.

Since 2007, three companies (Navigenics in Redwood Shores, California, deCODE Genetics of Reykjavik, Iceland, and 23andMe in Mountain View, California) have each been offering direct-to-consumer, whole-genome testing for markers thought to be predictive of traits and disorders ranging from the ability to roll your tongue to chronic diseases like cancer and coronary artery disease. To do these tests, the companies examine the DNA from a customer's saliva sample (via a convenient mail-in kit) at up to a million of the sites of genetic variation known as single nucleotide polymorphisms. The customer is delivered an individualized report of predicted health risks, based on aggregate data from hundreds of single-gene and genome-wide association studies on various traits and diseases. Many customers are apparently satisfied with the services they receive. However, a dilemma precipitates when customers face ambiguous or alarming results needing expert interpretation, and they heed the timeless suggestion: ask your health care provider for more information.

Data suggest that when faced with such a dilemma, the American public will turn to their primary care providers (PCPs). In one study of 1,000 individuals living in the United States, 72% of respondents indicated that they would ask their PCP if they had a question about genetics [1]. Indeed, based on personal experience at Stanford University Hospital & Clinics, situated at the nexus of the genomics revolution in Silicon Valley, patients have already begun to arrive at their physician's office with 23andMe reports in hand seeking expert medical advice. While some physicians are equipped to interpret such reports, evidence indicates that the majority of physicians are poorly prepared to deal with issues related to genetics and genomics, and that such patients are likely to be disappointed and misinformed [2]–[4]. In one survey of 5,915 individuals and families with genetic conditions, 64% of respondents reported receiving no genetics education materials from the health care provider they named most important in the management of their condition [4]. Another study showed that one-third of physicians incorrectly interpreted the results of a single-gene test for colorectal cancer susceptibility [5]. Even educational material mailed to practicing physicians by a genetic testing company proved ineffective in one study, where each basic question about genetic testing was answered incorrectly by over half of physicians, irrespective of receipt of the educational mailer [6]. This suggests more formal and fundamental training is required to enhance physicians' knowledge about genetics and genetic testing, and their ability to provide effective genetic counseling; indeed, 68% of physicians in the study reported an increased need or desire to learn more about genetics and genetic testing. Finally, physicians often fail to elicit detailed or updated family histories on their patients, an omission several studies have found to hinder the effective use of genetic testing and genetic counseling [7],[8]. Together, these studies suggest that many physicians are not adequately trained to appropriately order, or interpret and communicate results from even single-gene tests, let alone tests incorporating the full genome.

Harnessing the potential power of genomics in medicine requires physicians who can effectively use genetic tests and critically evaluate and interpret their results. The paucity of such physicians reflects the lack of sufficient education in genetics and genomics throughout medical training. As genetic components underlie disorders in nearly all medical domains, and patients most often turn to their PCP for questions about genetic testing [1], all medical trainees should be well-versed in the basic principles of genetics and genomics. Moreover, with only 3,300 genetics professionals in the United States who are certified by the American Board of Medical Genetics and/or the American Board of Genetic Counseling [9], PCPs will be increasingly relied upon to perform genetic counseling and risk assessment. Without appropriate revisions in clinician training, the supply of genomics-savvy physicians could be markedly outstripped by a rapidly increasing demand.

## Closing the Gap in Medical Education

The genetics education that physicians-in-training typically receive in medical school and graduate medical education is ill-suited for practicing personalized medicine. Medical schools should improve their curricula to include not only more basic science concepts in genetics and genomics, but also practical training for their applications in clinical medicine. Unfortunately, in a recent study only 11% of US and Canadian medical schools reported practical training in the use of medical genetics as part of their curricula [10]. A natural starting place to instill the essential core competencies is the first-year medical school curriculum.

For example, the most basic skill with which every medical student should be invested is how to take a “genetic history”—that is, a family history of adequate detail and accuracy to assess genetic risk. Coupled with this requirement, every medical student should also learn how to synthesize this information to determine the clinical contexts where genetic/genomic testing or referral to a genetic counselor is warranted. To achieve these learning objectives, medical schools should strive to teach genetics and genomics beyond the scope of a stand-alone basic science course. Genetics should be incorporated into a “Practice of Medicine” or equivalent course, where medical students learn how to conduct the patient interview and physical examination. Equally important, improved education is needed to enable medical trainees to interpret the results of genetic tests in a clinical setting.

To further prepare for the dawning era of personalized medicine, medical students must learn the principles of genetic variation in the human population and how genome-wide studies of complex diseases are conducted and analyzed. Notably, the same principles from simple Mendelian genetics cannot be applied to the genomics of complex diseases (e.g., diabetes, cancer, asthma, and heart disease). Genome-wide association studies often find genetic variants that contribute very little to overall risk for disease and are statistically determined at a population level, which can be misleading when applied directly to an individual. For example, a man who learns he has a low-risk genetic variant for heart disease (e.g., a single nucleotide polymorphism with an odds ratio of 0.90, or a decreased risk of 10%) should not in turn disrupt his exercise or diet regimen, as the protective genetic effect may be easily offset by the deleterious effect of an unhealthy lifestyle. If the variant is common in the population, its effect may be statistically significant at the population level, but not clinically significant for an individual patient. The reverse may hold in other cases, where genetic risk trumps environmental risks. For example, a patient harboring a variant of the *APOE* gene has anywhere between a 6-fold and 33-fold increased risk of developing Alzheimer disease, depending on the individual's race [11]. Genetic variation in the population also underlies patient-to-patient differences in drug response. Underscoring the importance of pharmacogenetics to clinical practice, the US Food and Drug Administration has recently modified the label for drugs such as warfarin and clopidogrel (Plavix) to warn that genetic variants present in a patient may warrant careful assessment of whether to use the drug and what dose to use. Finally, for many complex disorders new risk-modulating genetic variants, and their interactions with each other and with environmental factors, continue to be discovered on an ongoing basis. Thus, medical students must be taught how genetic factors for disease and drug response are determined, how they are modified by other factors, and how to interpret the significance of test results in the context of an individual patient with a specific medical profile.

Several national organizations are calling for improved genetics education among health professionals. To help drive and coordinate this effort in the United Kingdom, the National Genetics Education and Development Centre (http://www.geneticseducation.nhs.uk/) has developed evidence-based learning objectives and competencies in genetics for health professionals [12]. In the United States, the National Coalition for Health Professional Education in Genetics (NCHPEG; http://www.nchpeg.org/) has also identified a set of core competencies in genetics that all health professionals should possess. The strategies offered by these organizations for improving genetics education will serve as a strong starting place for medical school and graduate medical education reform.

## Conclusion

As genetic and genomic health information become routine components of patients' health records, we must as physicians become facile with the use and interpretation of this information as early as possible in our careers. Fundamental training in genetics and genomics, along with the attendant medical, legal, ethical, and psychosocial issues, should fall within the purview of medical school education. Moreover, such education should continue longitudinally throughout clinical training, including residency, fellowship, and continuing medical education programs, to reinforce concepts and target physicians who have already completed their clinical training. The potential of genetics and genomics to provide new paradigms for prevention, diagnosis, and treatment of disease is immense, but before the vision of a personalized medicine can be fully realized, medical trainees must be given the proper educational foundation.

## Abbreviations

PCP: primary care provider
